# Development and Internal Validation of a Machine Learning-Based Model for Thalassemia Identification Using Complete Blood Count Parameters

**DOI:** 10.3390/diagnostics16172815

**Published:** 2026-09-01

**Authors:** Ping Yin, Jialin Tan, Honghai Hong

**Affiliations:** Department of Clinical Laboratory, Guangdong Provincial Key Laboratory of Major Obstetric Diseases, Guangdong Provincial Clinical Research Center for Obstetric and Gynecology, The Third Affiliated Hospital, Guangzhou Medical University, Guangzhou 510150, China

**Keywords:** thalassemia, anemia, complete blood count, machine learning, random forest

## Abstract

**Background/Objectives:** While genetic testing for thalassemia is definitive yet costly for population screening, and conventional methods lack specificity, this study aims to develop and internally validate a machine learning (ML) model based on routine complete blood count (CBC) parameters to identify individuals at increased risk of thalassemia who may benefit from confirmatory genetic testing. **Methods:** Data from 1820 individuals were retrospectively collected and randomly divided using stratified sampling into training (70%) and internal validation (30%) cohorts. Feature selection was conducted within the training cohort, reducing 225 analyzer-derived parameters to 12 features. SMOTE was applied to the training portion of each five-fold cross-validation split. Seven ML models were evaluated in the validation cohort, with post hoc global feature attribution assessed using SHAP. **Results:** Seven ML models were developed using 12 selected features. All performed strongly (AUC: 0.885–0.918), with Random Forest (RF) achieving the highest AUC of 0.918 (95% CI: 0.900–0.940), accuracy of 85.7%, sensitivity of 89.7%, specificity of 83.0%, and F1 score of 0.837, with a PPV of 78.4% and NPV of 92.1%. SHAP analysis identified mean corpuscular volume (MCV), mean corpuscular hemoglobin (MCH), and red blood cell distribution width-standard deviation (RDW-SD) as the most influential features. **Conclusions:** The RF-based model showed good discrimination in validation, while SHAP analysis provided information on global feature contributions. The model may assist in identifying individuals who warrant confirmatory testing; however, prospective external validation is required before routine clinical implementation.

## 1. Introduction

Thalassemia is an inherited hemolytic anemia caused by defects in globin genes [1], with a well-documented high prevalence in regions such as the Mediterranean, the Middle East, Southeast Asia, and southern China [2,3]. Carriers of thalassemia typically remain asymptomatic, yet the genetic risk can lead to the birth of children with thalassemia major, imposing a significant health and economic burden on families and society, which includes lifelong transfusion dependence, iron chelation therapy, and the associated high costs and reduced quality of life for patients [4]. Consequently, widespread carrier screening coupled with genetic counseling is a crucial strategy. It not only prevents the birth of thalassemia major cases but also informs tailored management strategies for affected individuals, ultimately alleviating the public health burden [5]. This preventive approach has been successfully implemented in several high-prevalence regions, such as Cyprus and Sardinia, leading to a dramatic reduction in the incidence of new thalassemia major births [6,7], thereby validating the strategy’s effectiveness.

The complete blood count (CBC), due to its high accessibility and low cost, serves as a primary screening tool. MCV and MCH provide an intuitive characterization of red blood cells (RBCs) as exhibiting microcytosis and hypochromia [3,8,9], thereby guiding clinicians in determining the necessity for additional diagnostic investigations. Beyond MCV and MCH, additional CBC parameters, including the red cell distribution width (RDW), also contribute to distinguish thalassemia [10,11,12]. However, the specificity of traditional thalassemia screening based only on these CBC parameters can be compromised by conditions such as iron deficiency anemia (IDA), which presents with microcytic hypochromic RBCs, creating a significant diagnostic overlap that leads to false positives in thalassemia screening and unnecessary referrals for costly confirmatory tests [13]. While genetic testing remains the gold standard for diagnosis, its high cost and technical requirements hinder its application in large-scale primary screening [14]. Established discriminant indices based on CBC parameters, such as the Mentzer index, fail to fully utilize the multi-parameter information of CBC and lack unified standardized thresholds [15]. Consequently, their diagnostic performance varies significantly across different studies [16].

In recent years, ML methods have emerged as a novel approach for the efficient utilization of CBC data [17], demonstrating the capability to improve various aspects of hematology analysis, ranging from cellular morphology recognition to diagnosis of disease and prognostic prediction [18,19,20]. Unlike rule-based formulas, ML algorithms leverage the full spectrum of CBC parameters by capturing complex nonlinear relationships among them, rather than relying on pre-defined thresholds or assumptions [21]. This data-driven approach captures complex relationships between variables, including both explicit associations and latent interactions, offering a pathway to superior clinical performance [22]. This capability facilitates the development of models that can be optimized for specific populations or generalized across diverse cohorts, thereby enhancing both their adaptability and diagnostic accuracy [17].

Previous studies have demonstrated the potential of machine-learning approaches for thalassemia screening using hematological parameters. For example, RF-based models have been developed to identify α-thalassemia carriers among individuals with low HbA_2_ and to differentiate thalassemia from iron deficiency anemia [23,24]. More recently, machine-learning models based on routine blood parameters have also been evaluated for distinguishing thalassemia trait from non-thalassemia individuals [25]. However, the clinical differentiation of thalassemia from a heterogeneous non-thalassemia population, including both patients with other forms of anemia and individuals with normal hematological status, remains challenging. Therefore, we included a substantial non-thalassemia anemia group as a clinically challenging negative population and developed machine-learning models based on CBC parameters. We hypothesized that integrating multiple hematological features through machine learning would improve discrimination and provide a better balance between sensitivity and specificity than conventional CBC-based screening rules. Accordingly, this study aimed to develop and internally validate a machine-learning model for identifying individuals at increased risk of thalassemia who may benefit from confirmatory genetic testing.

## 2. Materials and Methods

### 2.1. Study Population

Records were retrospectively collected from individuals who underwent thalassemia genetic testing at the Third Affiliated Hospital of Guangzhou Medical University between May 2024 and September 2025. Genetic testing had been performed as part of routine thalassemia carrier screening or clinically indicated diagnostic evaluation. The inclusion criteria were as follows: (1) age ≥ 18 years; (2) availability of thalassemia genetic testing results; (3) complete records of age, sex, and CBC parameters; and (4) eligibility for classification into one of the three predefined study groups. Participants were excluded if they met any of the following criteria: (1) acute bleeding; (2) ongoing treatment for thalassemia before CBC testing; (3) indeterminate clinical diagnosis; or (4) analyzer-, sample-, or instrument-related quality abnormalities, including analyzer alarms, abnormal background signals, or sample aspiration errors.

The dataset was assembled to include three prespecified groups: thalassemia, non-thalassemia anemia, and normal controls. Participants were not consecutively enrolled; records were retrospectively selected according to the predefined study-group criteria. The thalassemia group included individuals with genetically confirmed thalassemia who were newly diagnosed and had not received treatment for thalassemia before CBC testing. The non-thalassemia anemia group included individuals with negative thalassemia genetic testing and a documented clinical diagnosis of another type of anemia in the Hospital Information System (HIS). These diagnoses were established by the treating physicians based on the available clinical and laboratory findings. Normal controls were defined as individuals with negative thalassemia genetic testing and normal hemoglobin levels (≥120 g/L in women and ≥130 g/L in men). For individuals with repeated records during the study period, only one record per person was retained.

The CBC test was performed by DH-800CS hematology analyzer (Dymind Biotechnology Co., Ltd., Shenzhen, China). The reverse dot blot hybridization (RDB) and Gap-polymerase chain reaction (Gap-PCR) methods (Yaneng BIOscience Co., Ltd., Shenzhen China) were employed to identify thalassemia gene mutations and deletions, respectively. For α-thalassemia, Gap-PCR was used to detect three common deletional variants: --^SEA^, -α^3.7^, and -α^4.2^, while RDB was used to detect three non-deletional variants: αα^CS^, αα^QS^, and αα^WS^. For β-thalassemia, RDB was used to detect 17 common mutations: CD41-42(-TCTT), IVS-II-654(C→T), CD17(A→T), -28(A→G), β^E^(G→A), CD71-72(+A), CD43(G→T), -29(A→G), Int(ATG→AGG), CD14-15(+G), CD27-28(+C), -32(C→A), -30(T→C), IVS-I-1(G→T), IVS-I-5(G→C), CD31(-C), and CAP+40–+43(-AAAC).

### 2.2. Ethical Approval

The ethical approval for the collection and utilization of all venous blood samples in this study was received from the Ethical Committee of Third Affiliated Hospital of Guangzhou Medical University (Approval no. [2024]197). The clinical and laboratory data generated were pre-existing medical records collected during routine clinical care and were subsequently accessed retrospectively for the study after ethics approval. No additional clinical procedures, examinations, or biological samples were performed or collected specifically for this study. As this was a retrospective study, the requirement for informed consent was waived. The study was performed in accordance with the ethical principles outlined in the Declaration of Helsinki.

### 2.3. Data Processing

Before model development, data cleaning and quality-control procedures were performed. First, 161 records were excluded because of acute bleeding, ongoing treatment for thalassemia, or an indeterminate diagnosis. Subsequently, an additional 81 records were excluded because of analyzer alarms or other sample- or instrument-related quality abnormalities, including abnormal background signals or sample aspiration errors. After these exclusions, 1820 records remained for subsequent analysis. The dataset was then stratified according to the three prespecified study groups (thalassemia, non-thalassemia anemia, and normal controls) and randomly divided into a training cohort (70%) and a validation cohort (30%) using a fixed random seed (random_state = 42). Z-score standardization was performed using the mean and standard deviation calculated from the training cohort, and the same parameters were subsequently applied to the validation cohort.

### 2.4. Feature Selection

Feature selection was performed within the training cohort. First, all hematological parameters generated by the analyzer were compared among the thalassemia, non-thalassemia anemia, and normal-control groups using statistical tests. Multiple testing was controlled using the Benjamini–Hochberg false discovery rate (FDR) procedure, and parameters with significant between-group differences after FDR correction were retained as candidate features. Among these statistically selected features, HGB, MCV, MCH, and MCHC were retained for subsequent model development because of their established clinical relevance in thalassemia screening, as supported by the Chinese Expert Consensus on Carrier Screening and Prevention of Thalassemia. To reduce redundancy among the remaining candidate features, hierarchical clustering was performed using Ward linkage, with the distance between variables defined as 1 − ∣ρ∣, where ρ denotes the Spearman rank correlation coefficient. A distance threshold of 0.30 was used to define feature clusters. The retained features were subsequently ranked according to Gini importance calculated using DecisionTreeClassifier from scikit-learn (version 1.5.2). To assess the potential model dependence of Gini-based feature ranking, mutual information was additionally applied as a model-agnostic feature-ranking method.

### 2.5. Model Construction and Comparison

For model development, genetically confirmed thalassemia cases were defined as the positive class, while the non-thalassemia anemia and normal-control groups were combined as the negative class. Thus, the final prediction task was binary classification of thalassemia versus non-thalassemia. We employed seven prevalent ML algorithms based on selected features to develop the models, among which were Logistic Regression (LR), Decision Tree (DT), Random Forest (RF), Gradient Boosting (GB), eXtreme Gradient Boosting (XGBoost), Support Vector Machine (SVM), and the Multi-Layer Perceptron (MLP).

Model hyperparameters and the classification threshold were determined within the training cohort using five-fold cross-validation. In each fold, Synthetic Minority Over-sampling Technique (SMOTE) was applied to the training portion, while the corresponding validation fold remained unresampled. As a sensitivity analysis, model performance was additionally evaluated without SMOTE to assess the influence of oversampling. Candidate hyperparameter configurations were evaluated across the five folds, and the configuration achieving the highest mean cross-validated AUC was selected as the optimal configuration. For this configuration, the optimal classification threshold was determined in each validation fold by maximizing the Youden index. The final classification threshold was then defined as the mean of the optimal thresholds across the five folds. Finally, the model was refitted using the full training cohort with the selected hyperparameters, with SMOTE applied to the training data. The resulting model and prespecified classification threshold were then applied without modification to the validation cohort.

Following the model development and threshold-selection strategy, the final classification thresholds were 0.498 for LR, 0.364 for DT, 0.548 for RF, 0.388 for GB, 0.413 for XGBoost, 0.612 for SVM, and 0.526 for MLP. Detailed hyperparameter configurations for each model are provided in the Supplementary Table S1.

Model performance was evaluated using eight metrics: AUC, accuracy, sensitivity, specificity, F1 score, PPV, NPV, and Brier score, supplemented by four analytical methods: Receiver Operating Characteristic (ROC) curve, calibration curve, confusion matrix analysis and decision curve analysis (DCA). Calibration reflects the concordance between a model’s predicted and actual disease risk. As such, calibration curves were employed as the primary visual tool for identifying and comparing calibration performance among the ML models [26]. Confusion matrix analysis was used to identify cases where the model’s predictions did not match the true outcomes. To assess the model’s potential for clinical implementation, DCA was conducted to quantify its net benefit across a spectrum of decision thresholds, thereby illustrating the trade-offs between clinical gains and intervention costs [27].

### 2.6. Model Evaluation and Explanation

Following comparison, the model exhibiting the highest AUC value on the validation cohort was selected for subsequent analysis. To assess its clinical utility, the diagnostic performance was compared against the conventional screening indices. The formulas and training-cohort–optimized cutoff values for the conventional screening indices are provided in Supplementary Table S2. For each screening index, the optimal cutoff was determined in the training cohort by maximizing the Youden index and was subsequently applied unchanged to the validation cohort. Accordingly, these cutoffs were data-optimized rather than prespecified. For the matched-sensitivity analysis, the RF threshold was adjusted post hoc to achieve a sensitivity closest to 97.3%. Post hoc model explanation was performed using SHAP TreeExplainer (version 0.42.1). SHAP values were calculated for the positive class (thalassemia), and global feature importance was summarized using the mean absolute SHAP value of each feature across the analyzed samples.

### 2.7. Statistical Analysis

Categorical data of the study were summarized as proportions and compared using the chi-square test. Continuous variables with a normal distribution are presented as mean ± SD. For between-group comparisons of continuous variables, parametric tests (independent samples *t*-test for two groups; one-way ANOVA for multiple groups) were used if data met assumptions of normality and homogeneity of variance. If these assumptions were violated, corresponding non-parametric tests were applied (Mann–Whitney U test or Kruskal–Wallis H test, respectively). Welch‘s *t*-test was specifically used for two-group comparisons when normality was satisfied but variances were unequal. Statistical significance was defined as *p*-value < 0.05. The 95% confidence intervals (CIs) for AUC, Brier score, and F1 score were estimated using nonparametric bootstrap resampling. For accuracy, sensitivity, specificity, PPV and NPV, 95% CIs were calculated using the Wilson score method. The AUCs of the RF and LR models were compared using a paired DeLong test. All analyses and model development were performed using Python version 3.11, with scikit-learn version 1.5.2, pandas version 1.2.4, NumPy version 1.20.1, matplotlib version 3.7.5, and SHAP version 0.42.1.

## 3. Results

### 3.1. Demographic and Baseline Characteristics

**Figure 1:**
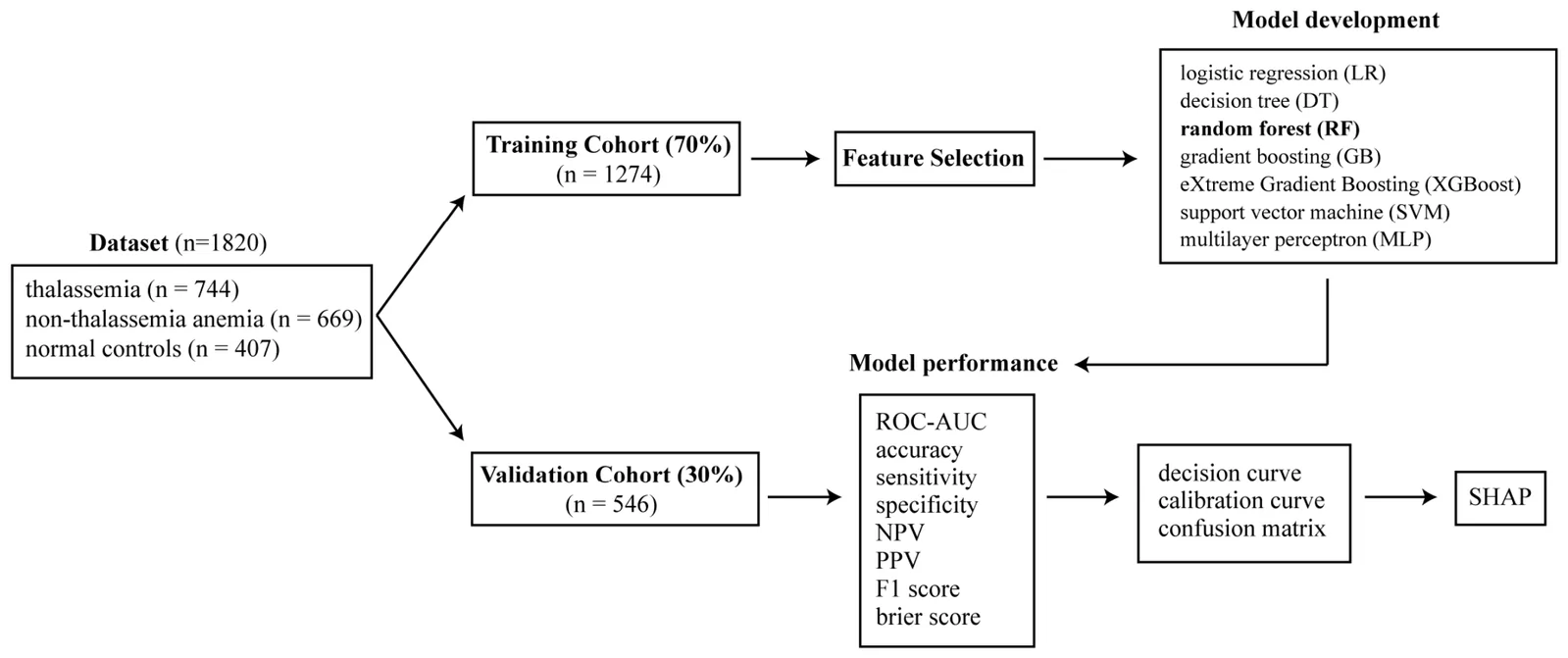
Flowchart of model development and validation.

A total of 1820 individuals who underwent thalassemia gene testing between May 2024 and September 2025 were included in this study. They were categorized into three groups: the thalassemia group (*n* = 744), the non-thalassemia anemia group (*n* = 669), and the normal control group (*n* = 407). The baseline characteristics of the participants are detailed in Table 1. All participants were randomly assigned in a 7:3 ratio, yielding a training cohort of 1274 individuals and a validation cohort of 546 individuals. Significant differences in baseline characteristics and hematological parameters were observed across the three groups (*p* < 0.05), but no significant differences were identified between the training cohort and the validation cohort (Table 2). A flow chart of our study design is illustrated in Figure 1.

### 3.2. Discriminability of the Models

A total of 225 parameters were obtained for each sample analyzed on the DH-800CS analyzer. Statistical screening performed in the training cohort identified 22 parameters with significant between-group differences, which were retained as candidate features (Supplementary Table S3). Among these, HGB, MCV, MCH, and MCHC were retained because of their established clinical relevance in thalassemia screening. The remaining candidate features were further evaluated using Spearman correlation-based hierarchical clustering to reduce redundancy and were subsequently ranked according to Gini importance (Supplementary Figure S1). This procedure resulted in a final set of 12 features for model development: RBC, HGB, MCV, MCH, MCHC, HCT, RDW-SD, RDW-CV, Micro%, Macro%, L-MCV, and S-MCV.

**Figure 2:**
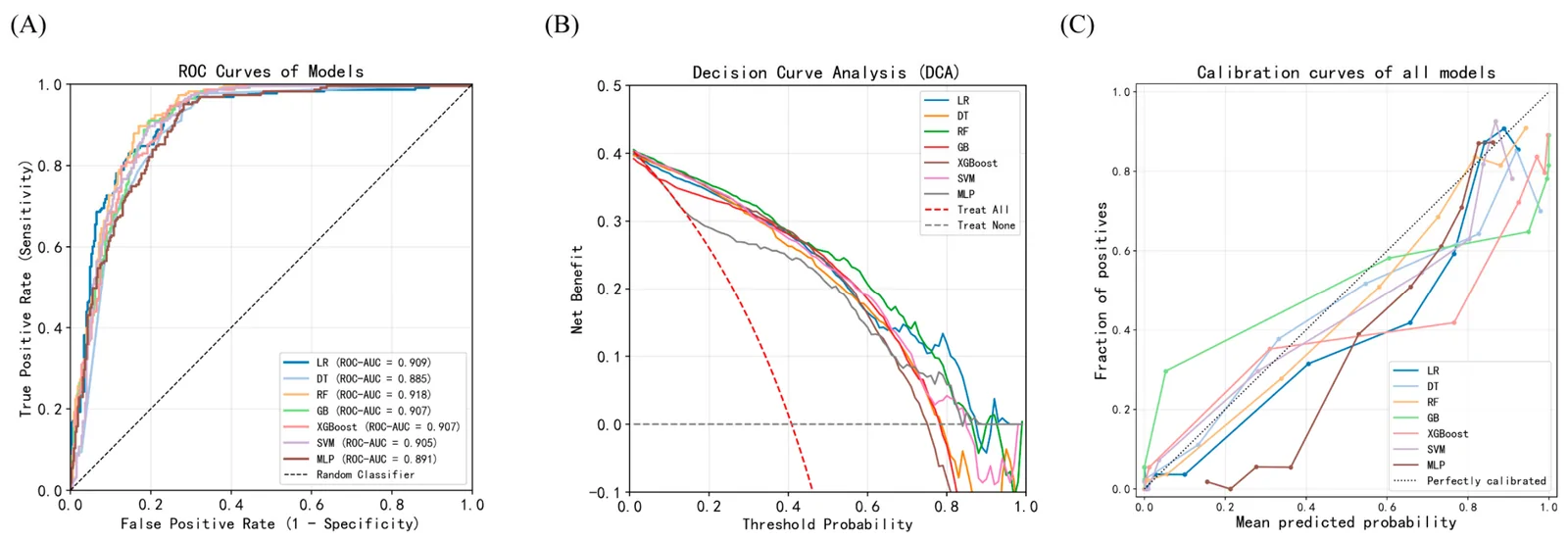
Model performance in the validation cohort. (**A**) Receiver operating characteristic (ROC) curves of the machine-learning models for thalassemia identification. (**B**) Decision curve analysis (DCA) of the models across threshold probabilities. (**C**) Calibration plots comparing predicted and observed probabilities in the validation cohort. LR, Logistic Regression; DT, Decision Tree; RF, Random Forest; GB, Gradient Boosting; XGBoost, eXtreme Gradient Boosting; SVM, Support Vector Machine; MLP, Multi-Layer Perceptron.

As summarized in Table 3 and Figure 2A, all models demonstrated strong performance in the validation cohort, with AUC values ranging from 0.885 to 0.918. Among the evaluated models, RF achieved the highest AUC of 0.918 (95% CI: 0.900–0.940), with an accuracy of 85.7% (95% CI: 82.5–88.4%), PPV of 78.4% (95% CI: 73.0–83.0%), sensitivity of 89.7% (95% CI: 85.0–93.0%), specificity of 83.0% (95% CI: 78.5–86.7%), F1 score of 0.837 (95% CI: 0.801–0.871), and NPV of 92.1% (95% CI: 88.4–94.7%). Furthermore, the F1 score was optimal for RF, indicating a balanced performance in precision and sensitivity. These results suggest that machine learning models, particularly RF, offer effective discriminative capability for thalassemia identification based on CBC parameters. Although RF achieved a higher observed AUC than LR (0.918 vs. 0.909), the difference was not statistically significant according to the paired DeLong test (*p* > 0.05).

In the sensitivity analysis without SMOTE, model performance remained broadly similar. RF achieved an AUC of 0.913 (95% CI: 0.893–0.937) and a Brier score of 0.111 (95% CI: 0.095–0.128), compared with 0.918 and 0.110, respectively, in the primary SMOTE-based analysis (Supplementary Table S4).

To further assess model portability, we developed a reduced RF model using only seven routinely available CBC parameters (RBC, HGB, MCV, MCH, MCHC, HCT, and RDW-CV). In the validation cohort, the reduced model achieved an AUC of 0.905 (95% CI: 0.884–0.929), sensitivity of 95.1%, specificity of 76.2%, and F1 score of 0.828. Compared with the full 12-feature RF model, the reduced model retained good discrimination while showing higher sensitivity but lower specificity. Detailed performance metrics are presented in Supplementary Table S5.

To evaluate model performance in a more clinically challenging setting, an additional analysis was performed after excluding normal controls and restricting the comparison to thalassemia versus non-thalassemia anemia. After excluding normal controls and applying the original fitted models and prespecified thresholds unchanged, RF achieved an accuracy of 81.6%, sensitivity of 89.7%, specificity of 72.6%, F1 score of 0.837, PPV of 78.4%, and NPV of 86.4%. Compared with the primary analysis, sensitivity, PPV, and F1 score remained unchanged, whereas accuracy (85.7% to 81.6%), specificity (83.0% to 72.6%), and NPV (92.1% to 86.4%) decreased, indicating greater difficulty in distinguishing thalassemia from other anemias. Detailed results for all models are presented in Supplementary Table S6.

### 3.3. Clinical Applicability of the Models

In line with TRIPOD + AI guidelines emphasizing clinical applicability [28], decision curve analysis (DCA) was used to assess the potential clinical utility of the models (Figure 2B). Given the intended use of the model as a screening tool to support referral for confirmatory testing, interpretation was focused on the clinically plausible low-to-moderate threshold probability range. Within this range, the RF model generally provided a positive net benefit relative to the treat-all and treat-none strategies. However, the decision curves of the ML models crossed, and no single model consistently achieved the highest net benefit across all thresholds. These findings suggest potential clinical utility of the RF model but do not demonstrate uniform superiority over the other ML models.

### 3.4. Calibration Performance of the Models

Calibration plots were used to visually assess the agreement between predicted and observed probabilities (Figure 2C). The RF model showed relatively close visual agreement with the ideal reference line. In addition, the RF model achieved a Brier score of 0.110 (95% CI: 0.094–0.128), indicating relatively good overall probabilistic prediction performance in the validation cohort. The Brier scores of all evaluated models are presented in Table 3.

### 3.5. Optimal Model Evaluation and Explanation

**Figure 3:**
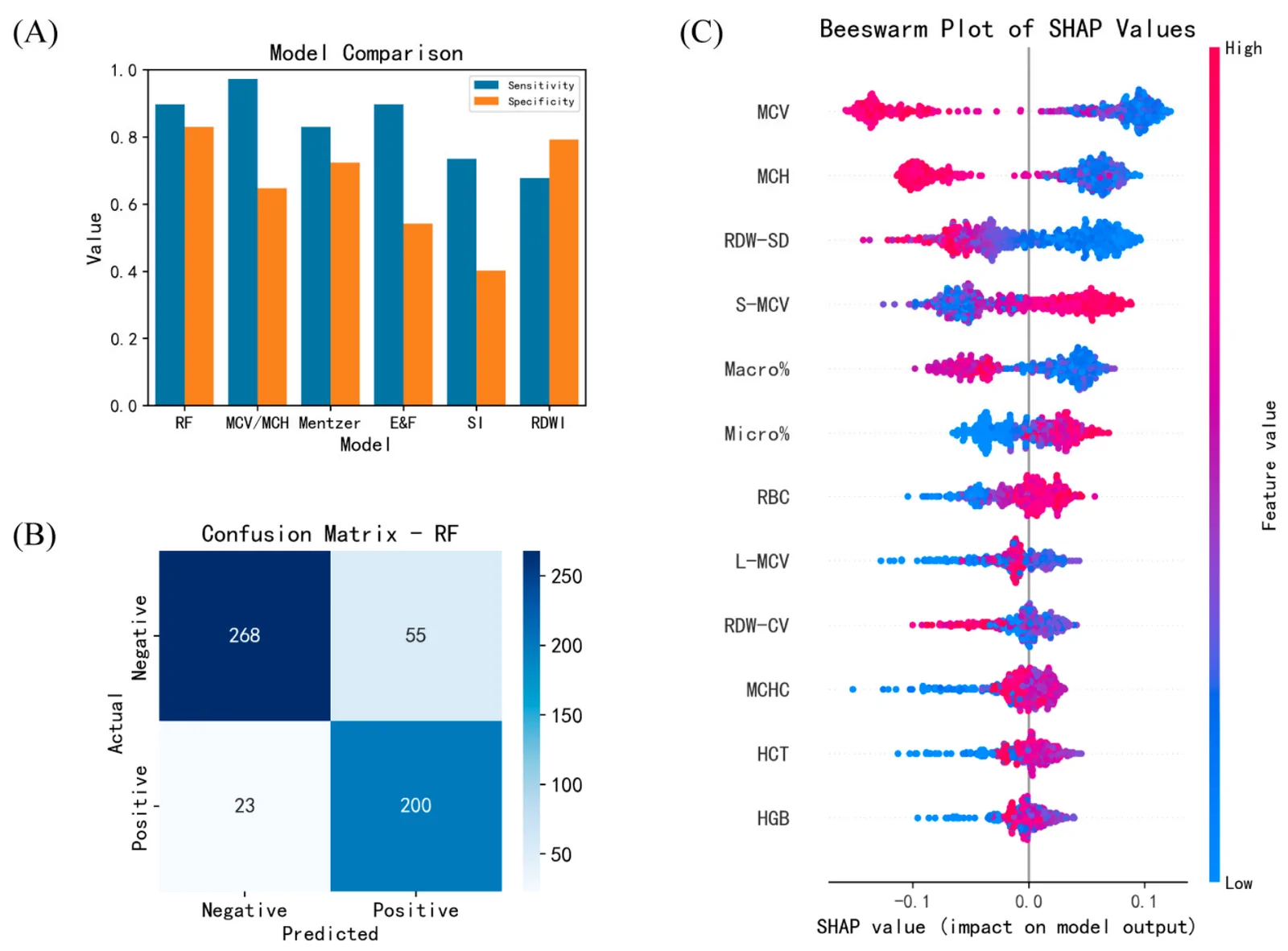
Optimal model evaluation and explanation. (**A**) Comparison of the sensitivity and specificity of the RF model and conventional thalassemia screening methods. MCV/MCH: MCV/MCH rule; Mentzer: Mentzer index; E&F: England and Fraser index; SI: Sirachainan index; RDWI: RDW index. (**B**) Confusion matrix of the RF model for thalassemia identification in the validation cohort. (**C**) Results of SHapley Additive exPlanations (SHAP) analysis of the RF model.

Based on its overall performance, including the highest observed AUC, accuracy, specificity, and F1 score, the RF model was selected for further evaluation. Consequently, we proceeded to a detailed evaluation of its identification efficacy using the validation cohort via confusion matrix analysis. The RF model was further compared with several conventional thalassemia screening approaches, including the MCV/MCH rule, Mentzer index, RDW Index, England and Fraser Index, and Sirachainan Index (Figure 3A). The RF model achieved a sensitivity of 89.7% and a specificity of 83.0%. In comparison, the MCV/MCH rule showed higher sensitivity (97.3%) but lower specificity (64.7%), indicating a trade-off between reducing false-positive referrals and avoiding missed thalassemia cases.

To enable a clinically relevant comparison at a matched high sensitivity, the RF model and conventional screening indices were further evaluated at a sensitivity of approximately 97.3%, corresponding to that of the MCV/MCH screening rule. At this operating point, the RF model retained a specificity of 73.1%, with a PPV of 71.4% and an NPV of 97.5%. Detailed comparisons are provided in Supplementary Table S7.

The analysis of the 23 false-negative cases revealed that 15 were silent carriers, 5 were minor thalassemia cases, and 3 cases with coexisting IDA. Among the 55 false-positive cases, 45 were from patients with anemia of chronic disease, and the remaining 10 were from patients with IDA (Figure 3B). To characterize the global contribution of individual features to RF predictions, SHAP analysis was performed. MCV, MCH, and RDW-SD showed the largest global feature contributions (Figure 3C).

## 4. Discussion

Advances in machine learning offer promising avenues for refining the diagnostic pathway for thalassemia. By leveraging routinely available clinical and laboratory data, machine learning models can potentially uncover complex, non-linear patterns that are difficult to discern through traditional rule-based methods [29]. Developing such tools holds the potential to augment clinical decision-making, particularly in scenarios requiring efficient screening or differentiation from other anemias, thereby optimizing resource allocation and improving patient management [30].

This study developed and internally validated a machine learning model based on CBC parameters to predict thalassemia status. Among the evaluated algorithms, RF achieved the highest observed AUC and favorable overall performance, likely because RF is an ensemble learning method based on the bagging principle. It constructs multiple decision trees independently during training and aggregates their predictions, effectively reducing model variance and enhancing generalization [31]. This approach makes it particularly robust against overfitting, especially in scenarios with complex feature interactions or moderate noise, which is often the case with clinical hematological data.

Additional analyses highlighted two important considerations regarding clinical applicability. First, model performance was attenuated when normal controls were excluded and the comparison was restricted to thalassemia versus non-thalassemia anemia, indicating that differentiation from other anemia disorders represents a more challenging clinical task. Second, a reduced RF model based only on routinely available CBC parameters retained good discrimination, although with lower specificity than the full model, suggesting a potential trade-off between cross-platform portability and predictive performance.

To characterize global feature contributions to RF predictions, SHAP analysis was performed, identifying MCV, MCH, and RDW-SD as the top three contributors to its decisions. Notably, the model’s dependence on MCV and MCH corroborates their central role in current thalassemia screening guidelines [32], while its identification of RDW-SD as a key feature supports the established clinical utility of RDW in differentiating between thalassemia and IDA [33].

Analysis of misclassified cases provided insights into the model’s limitations. Among the 23 false-negative cases, the majority (*n* = 15) were silent carriers, with an additional 5 cases of thalassemia minor; their CBC parameters showed minimal deviation from normal ranges due to attenuated phenotypic expression. The remaining 3 false-negatives occurred in patients with concurrent IDA, where the characteristic thalassemic hematological signature was likely masked. Conversely, the 55 false-positive results were driven by the presence of other microcytic anemias. The majority (*n* = 45) occurred in patients with anemia of chronic disease, a condition known to cause microcytosis, while the remainder (*n* = 10) were from patients with confirmed IDA, as both conditions share key hematological signatures with thalassemia.

The innovation of this study is reflected in three key aspects. First, the study was conducted in Guangdong Province, which has the highest incidence of thalassemia in China. Second, an interference group comprising 669 non-thalassemia anemia samples was included, which helps improve the model’s ability to differentiate thalassemia from other forms of anemia. Third, based on hematological parameters, SHAP analysis was incorporated to improve model transparency by identifying the features that contributed most strongly to predictions at the cohort level. However, several CBC parameters are correlated. SHAP attribution among correlated features may not be unique; therefore, individual SHAP values should be interpreted as model-specific feature attributions rather than independent or causal effects.

Several limitations of this study should be acknowledged. First, the model was developed for binary identification of thalassemia and does not distinguish specific genetic subtypes. In addition, the model did not specifically evaluate clinically important subgroups, including patients with thalassemia accompanied by iron deficiency or folate deficiency and individuals with double heterozygous genotypes. Furthermore, because the best-performing model was selected based on comparisons within the same internal validation cohort, some optimism in model selection cannot be excluded. Independent external validation or nested resampling approaches are warranted. Second, a major limitation is the single-center retrospective design. The cohort was derived from a tertiary hospital in southern China, and all participants had undergone thalassemia genetic testing. Consequently, the study population represented a selected referral cohort with a relatively high proportion of thalassemia cases and substantial differences in age and sex among the study groups. In addition, the specific anemia diagnoses within the non-thalassemia group were based on HIS-recorded clinical diagnoses and were not independently re-adjudicated using study-specific criteria, which may have introduced diagnostic heterogeneity within this clinically important comparison group. Because PPV and NPV are prevalence-dependent, the predictive values observed in this outcome-enriched cohort should not be directly extrapolated to lower-prevalence screening populations. As disease prevalence decreases, PPV would be expected to decrease, whereas NPV would generally increase. In addition, the mutation spectrum of thalassemia in southern China may differ from that in other geographic and ethnic populations, potentially resulting in population-specific hematological patterns. Although age and sex were not included as model features, these demographic, referral-related, and regional genetic characteristics may limit the generalizability of the model and the transferability of the observed decision-curve benefits to lower-prevalence or genetically different populations.

## 5. Conclusions

This study developed and compared seven machine learning models based on CBC parameters for thalassemia identification. Among the evaluated models, RF achieved the highest observed AUC and favorable overall performance in the present validation cohort, suggesting that routinely available hematological parameters may support the identification of individuals at increased risk of thalassemia. The model may potentially assist in prioritizing individuals for confirmatory testing; however, its clinical utility, generalizability, and impact on downstream testing and healthcare resource use require prospective external validation before implementation in routine practice.

## Figures and Tables

**Table 1:** Demographic characteristics and hematological parameters of three groups.

Variable	Thalassemia (*n* = 744)	Non-Thalassemia Anemia (*n* = 669)	Normal Controls (*n* = 407)	*p* Value
Sex				<0.001 ^a^
Male	108 (14.52%)	205 (30.64%)	197 (48.40%)	
Female	636 (85.48%)	464 (69.36%)	210 (51.60%)	
Age (years)	33 (30, 37)	52 (36, 71)	40 (30, 57)	<0.001 ^b^
RBC (10^12^/L)	5.11 (4.63, 5.65)	3.42 (2.49, 4.98)	4.50 (4.24, 4.86)	<0.001 ^b^
HGB (g/L)	112 (101, 121)	86 (71, 108)	138 (128, 148)	<0.001 ^b^
MCV (fL)	67.0 (64.0, 70.3)	79.8 (66.8, 93.1)	91.0 (88.5, 94.2)	<0.001 ^b^
MCH (pg)	21.5 (20.5, 22.7)	26.0 (21.0, 30.7)	30.4 (29.6, 31.4)	<0.001 ^b^
MCHC (g/L)	323 (318, 328)	322 (314, 331)	333 (329, 338)	<0.001 ^b^
HCT (%)	34.7 (31.5, 37.5)	26.8 (22.1, 33.5)	41.3 (38.6, 44.2)	<0.001 ^b^
Micro% (%)	33.1 (22.0, 45.0)	8.5 (1.9, 35.0)	0.9 (0.7, 1.3)	<0.001 ^b^
Macro% (%)	2.7 (2.1, 3.4)	4.3 (2.5, 7.2)	6.7 (6.1, 7.2)	<0.001 ^b^
S-MCV (fL)	46.3 (45.4, 46.9)	43.5 (42.3, 45.3)	44.2 (43.0, 45.1)	<0.001 ^b^
L-MCV (fL)	121.1 (119.5, 123.1)	120.8 (118.2, 125.1)	133.2 (131.7, 134.6)	<0.001 ^b^
RDW-CV (%)	16.2 (15.5, 17.1)	17.1 (15.6, 19.2)	13.6 (13.2, 14.1)	<0.001 ^b^
RDW-SD (fL)	38.5 (37.0, 40.5)	46.7 (40.9, 54.9)	43.5 (42.0, 45.4)	<0.001 ^b^

Abbreviations: RBC, red blood cell count; HGB, hemoglobin; MCV, mean corpuscular volume; MCH, mean corpuscular hemoglobin; MCHC, mean corpuscular hemoglobin concentration; HCT, hematocrit; Micro%, percentages of microcytes, Macro%, percentages of macrocytes, S-MCV, small red cell mean corpuscular volume; L-MCV, large red cell mean corpuscular volume; RDW-CV, red cell distribution width-coefficient of variation; RDW-SD, red cell distribution width-standard deviation. ^a^ chi-square test; ^b^ Kruskal–Wallis H test.

**Table 2:** Demographic characteristics and hematological parameters of the different cohorts.

Variable	Training Cohort (*n* = 1274)	Validation Cohort (*n* = 546)	*p* Value
Sex			0.178 ^a^
Male	360 (28.26%)	150 (27.47%)	
Female	914 (71.74%)	396 (72.53%)	
Age (years)	37 (31, 55)	36 (30, 50)	0.179 ^b^
RBC (10^12^/L)	4.63 (3.90, 5.25)	4.71 (4.06, 5.32)	0.071 ^b^
HGB (g/L)	112 (91, 127)	113 (94, 127)	0.197 ^b^
MCV (fL)	74.4 (66.2, 90.7)	72.3 (65.8, 89.7)	0.165 ^b^
MCH (pg)	23.9 (21.1, 30.1)	23.3 (21.0, 29.7)	0.165 ^b^
MCHC (g/L)	325 (318, 332)	325 (319, 332)	0.960 ^b^
HCT (%)	34.6 (28.1, 39.0)	35.1 (29.4, 39.1)	0.178 ^b^
Micro% (%)	13.4 (1.4, 36.5)	17.6 (1.6, 38.0)	0.237 ^b^
Macro% (%)	3.9 (2.5, 6.5)	3.7 (2.5, 6.3)	0.385 ^b^
S-MCV (fL)	45.0 (43.2, 46.3)	45.1 (43.2, 46.4)	0.293 ^b^
L-MCV (fL)	122.1 (119.6, 129.5)	122.5 (119.6, 130.1)	0.637 ^b^
RDW-CV (%)	16.0 (14.3, 17.3)	16.1 (14.4, 17.3)	0.525 ^b^
RDW-SD (fL)	41.8 (38.4, 46.2)	41.6 (38.5, 45.8)	0.608 ^b^

Abbreviations: RBC, red blood cell count; HGB, hemoglobin; MCV, mean corpuscular volume; MCH, mean corpuscular hemoglobin; MCHC, mean corpuscular hemoglobin concentration; HCT, hematocrit; Micro%, percentages of microcytes, Macro%, percentages of macrocytes, S-MCV, small red cell mean corpuscular volume; L-MCV, large red cell mean corpuscular volume; RDW-CV, red cell distribution width-coefficient of variation; RDW-SD, red cell distribution width-standard deviation. ^a^ chi-square test; ^b^ Mann–Whitney U test.

**Table 3:** The performance of ML for thalassemia identification in the validation cohort.

Model	AUC (95% CI)	Accuracy (95% CI)	Sensitivity (95% CI)	Specificity (95% CI)	F1 Score (95% CI)	PPV (95% CI)	NPV (95% CI)	Brier Score (95% CI)
LR	0.909 (0.884–0.931)	0.830 (0.796–0.859)	0.937 (0.897–0.962)	0.755 (0.706–0.799)	0.818 (0.782–0.851)	0.726 (0.671–0.774)	0.946 (0.911–0.967)	0.122 (0.104–0.141)
DT	0.885 (0.854–0.914)	0.808 (0.773–0.839)	0.933 (0.892–0.959)	0.721 (0.670–0.767)	0.798 (0.761–0.833)	0.698 (0.644–0.747)	0.940 (0.903–0.963)	0.133 (0.113–0.155)
**RF**	**0.918** **(0.900–0.940)**	**0.857** **(0.825–0.884)**	**0.897** **(0.850–0.930)**	**0.830** **(0.785–0.867)**	**0.837** **(0.801–0.871)**	**0.784** **(0.730–0.830)**	**0.921** **(0.884–0.947)**	**0.110** **(0.094–0.128)**
GB	0.907 (0.889–0.930)	0.848 (0.815–0.876)	0.910 (0.866–0.941)	0.805 (0.758–0.844)	0.830 (0.792–0.864)	0.763 (0.709–0.810)	0.929 (0.892–0.953)	0.139 (0.114–0.166)
XGBoost	0.907 (0.888–0.929)	0.832 (0.798–0.861)	0.946 (0.908–0.969)	0.752 (0.702–0.796)	0.821 (0.785–0.855)	0.725 (0.671–0.773)	0.953 (0.920–0.973)	0.139 (0.115–0.163)
SVM	0.905 (0.881–0.930)	0.842 (0.810–0.871)	0.897 (0.850–0.930)	0.805 (0.758–0.844)	0.823 (0.785–0.857)	0.760 (0.705–0.808)	0.919 (0.881–0.945)	0.123 (0.104–0.143)
MLP	0.891 (0.863–0.911)	0.813 (0.778–0.844)	0.951 (0.914–0.972)	0.718 (0.667–0.765)	0.806 (0.767–0.839)	0.700 (0.646–0.749)	0.955 (0.921–0.975)	0.154 (0.139–0.170)

Abbreviations: LR, Logistic regression; DT, Decision Tree; RF, Random Forest; GB, Gradient Boosting; XGBoost, eXtreme Gradient Boosting; SVM, Support Vector Machine; MLP, Multi-Layer Perceptron.

## Data Availability

The data generated and analyzed in this study are not publicly available due to patient privacy and ethical restrictions. The analysis code used for data preprocessing, model development, evaluation, and post hoc explanation will be made publicly available on GitHub after publication.

## Supplementary Materials

The following supporting information can be downloaded at: https://www.mdpi.com/article/10.3390/diagnostics16172815/s1, Figure S1: Correlation analysis and feature-importance ranking during feature selection; Table S1: Hyperparameter search spaces, selected hyperparameters, and model-specific random seeds for the seven machine-learning models; Table S2. Conventional screening indices and training-cohort–optimized cutoff values; Table S3. Initial statistical screening of hematological parameters in the training cohort; Table S4. Performance of machine-learning models without SMOTE in the validation cohort; Table S5. Performance of the reduced Random Forest model based on routinely available CBC parameters in the validation cohort; Table S6. Performance of machine-learning models for distinguishing thalassemia from non-thalassemia anemia after exclusion of normal controls.; Table S7. Performance of the RF model and conventional screening indices at a matched sensitivity level of approximately 97.3%.
