# Supplementary material for: Development and Internal Validation of a Machine Learning-Based Model for Thalassemia Identification Using Complete Blood Count Parameters
Source: Diagnostics (Basel). 2026 Sep 1;16(17):2815. doi: 10.3390/diagnostics16172815 (PMC13564637; doi:10.3390/diagnostics16172815)
Supplement: Supplementary file 1 [file diagnostics-16-02815-s001.zip › diagnostics-4437931-supplementary.pdf]

Table S1. Hyperparameter search spaces, selected hyperparameters, and model-specific random seeds for the seven machine-learning models.

Model	Hyperparameter search space	Final selected hyperparameters
LR	C: [0.001, 0.01, 0.1, 1,10, 100]	C: 1
	penalty: ['l1', 'l2']	penalty: 'l2'
	solver: ['liblinear','saga']	solver: 'saga'
DT	criterion: ['gini', 'entropy']	criterion: 'gini'
	max_depth: [5, 8, 10, 15, None]	max_depth: 8
	min_samples_split: [2, 3, 5, 7, 9]	min_samples_split: 5
	min_samples_leaf: [2, 3, 5, 7, 9]	min_samples_leaf: 9
	max_features: ['sqrt', 'log2', None]	max_features: 'sqrt'
RF	n_estimators: [100, 150, 200, 300,500]	n_estimators: 100
	max_depth: [5, 8, 12, 15, None]	max_depth: None
	min_samples_split: [2, 3, 5, 7, 9]	min_samples_split: 5
	min_samples_leaf: [2, 3, 5, 7, 9]	min_samples_leaf: 2
	max_features: ['sqrt', 'log2']	max_features: 'log2'
	bootstrap: [True, False]	bootstrap: True
	min_impurity_decrease: [0.0, 0.001, 0.01]	min_impurity_decrease: 0.0
GB	n_estimators: [25,50, 100, 200]	n_estimators: 200
	learning_rate: [0.01, 0.05, 0.1, 0.2]	learning_rate: 0.2
	max_depth: [3, 4, 5, 6]	max_depth: 6
	min_samples_split: [2, 5, 10]	min_samples_split: 2
	min_samples_leaf: [1, 2, 4]	min_samples_leaf: 4
	subsample: [0.8, 0.9, 1.0]	subsample: 0.8
	max_features: ['sqrt', 'log2', None]	max_features: 'log2'
	loss: ['log_loss']	loss: 'log_loss'
	criterion: ['friedman_mse', 'squared_error']	criterion: 'squared_error'
XGBoost	n_estimators: [25, 50, 100, 200]	n_estimators: 200
	learning_rate: [0.01, 0.05, 0.1, 0.2]	learning_rate: 0.1
	max_depth: [3, 4, 5, 6]	max_depth: 6
	min_child_weight: [1, 3, 5, 7]	min_child_weight: 1
	subsample: [0.6, 0.8, 1.0]	subsample: 0.6
	colsample_bytree: [0.6, 0.8, 1.0]	colsample_bytree: 0.8
	gamma: [0, 0.1, 0.5, 1]	gamma: 0
	reg_alpha: [0, 0.1, 1, 10]	reg_alpha: 1
	reg_lambda: [0.1, 1, 10]	reg_lambda: 0.1
	scale_pos_weight: [1, 5, 10]	scale_pos_weight: 5
SVM	C: [0.1, 1, 3, 5, 7,9]	C: 9
	kernel: ['rbf', 'poly', 'sigmoid']	kernel: 'rbf'
	gamma: ['scale', 'auto', 0.001, 0.01, 0.1, 1]	gamma: 'auto'
	degree: [2, 3]	degree: 3
MLP	hidden_layer_sizes: [(16,),(32,),	hidden_layer_sizes: [(16, 16)]
	(64,),(16,16),(32,16),(32,32),(32,64),(64,64)]	alpha: 0.0001

alpha: [0.0001, 0.001, 0.01]	learning_rate: 'adaptive'
learning_rate: ['constant', 'adaptive']	activation: 'tanh'
activation: ['relu', 'tanh']	

Random seed = 42 for all seven models.

Abbreviations: LR, Logistic regression; DT, Decision Tree; RF, Random Forest; GB, Gradient Boosting; XGBoost, eXtreme Gradient Boosting; SVM, Support Vector Machine; MLP, Multi-Layer Perceptron.

Table S2. Conventional screening indices and training-cohort–optimized cutoff values.

Screening method	Formula / positive criterion
MCV/MCH rule	MCV ≤ 82.22 and MCH ≤ 28
Mentzer index	MCV/RBC ≤ 16.16
RDW index	MCV × RDW-CV/RBC ≤ 232.77
England and Fraser index	MCV - RBC -5*Hb-3.4 ≤ 14.57
Sirachainan index	1.5*Hb - 0.05*MCV > 12

Abbreviations: MCV, mean corpuscular volume; MCH, mean corpuscular hemoglobin; RBC, red blood cell count; HGB, hemoglobin; RDW-CV, red blood cell distribution width–coefficient of variation.

Table S3. Initial statistical screening of hematological parameters in the training cohort.

Feature	Kruskal–Wallis P value	FDR-adjusted P value	Retained
HGB	3.25×10^{-142}	7.81×10^{-141}	Yes
Micro%	7.25×10^{-141}	8.70×10^{-140}	Yes
L-MCV	6.02×10^{-133}	4.81×10^{-132}	Yes
HCT	1.17×10^{-130}	7.02×10^{-130}	Yes
Micro#	6.52×10^{-129}	3.13×10^{-128}	Yes
MCV	3.06×10^{-120}	1.22×10^{-119}	Yes
R-MFV	1.88×10^{-118}	6.44×10^{-118}	Yes
RDW-CV	1.18×10^{-117}	3.55×10^{-117}	Yes
MCH	5.87×10^{-115}	1.57×10^{-114}	Yes
Macro%	1.07×10^{-111}	2.57×10^{-111}	Yes
Macro#	6.23×10^{-108}	1.36×10^{-107}	Yes
RDW-SD	2.36×10^{-102}	4.73×10^{-102}	Yes
S-MCV	8.58×10^{-92}	1.58×10^{-91}	Yes
RBC	1.54×10^{-72}	2.64×10^{-72}	Yes
MCHC	4.21×10^{-58}	6.31×10^{-58}	Yes
P-LCC	8.57×10^{-47}	1.21×10^{-46}	Yes
PCT	1.27×10^{-30}	1.70×10^{-30}	Yes
PDW	2.27×10^{-26}	2.87×10^{-26}	Yes
PLT-I	1.61×10^{-20}	1.85×10^{-20}	Yes
P-LCR	1.47×10^{-14}	1.60×10^{-14}	Yes

RBC_KURTOSIS	1.22 $\times 10^{-8}$	1.27 $\times 10^{-8}$	Yes
MPV	6.65 $\times 10^{-5}$	6.65 $\times 10^{-5}$	Yes

Abbreviations: FDR, false discovery rate; HGB, hemoglobin; HCT, hematocrit; MCV, mean corpuscular volume; RDW-CV, red blood cell distribution width–coefficient of variation; MCH, mean corpuscular hemoglobin; RDW-SD, red blood cell distribution width–standard deviation; RBC, red blood cell count; MCHC, mean corpuscular hemoglobin concentration; PCT, plateletcrit; PDW, platelet distribution width; MPV, mean platelet volume. Analyzer-specific parameter names are reported according to the DH-800CS output.

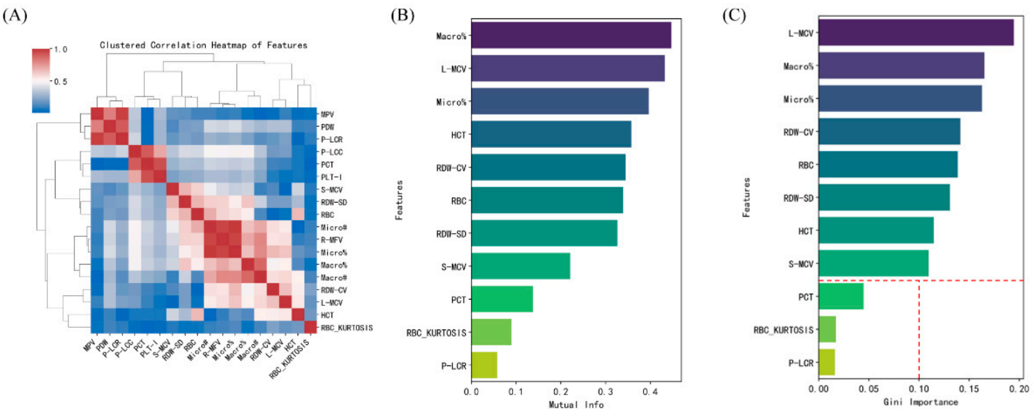

Figure S1. Correlation analysis and feature-importance ranking during feature selection. (A) Hierarchical clustering heatmap of the 18 candidate features remaining after the four clinically established CBC variables (HGB, MCV, MCH, and MCHC) were retained. (B) Feature ranking based on mutual information. (C) Feature ranking based on Gini importance.

Table S4. Performance of machine-learning models without SMOTE in the validation cohort.

Model	AUC (95% CI)	Accuracy (95% CI)	Sensitivity (95% CI)	Specificity (95% CI)	F1 score (95% CI)	PPV (95% CI)	NPV (95% CI)	Brier score (95% CI)
LR	0.909 (0.884-0.932)	0.832 (0.798-0.861)	0.933 (0.892-0.959)	0.762 (0.712-0.805)	0.819 (0.783-0.852)	0.730 (0.675-0.778)	0.943 (0.907-0.965)	0.122 (0.104-0.141)
DT	0.884 (0.851-0.911)	0.846 (0.813-0.874)	0.874 (0.825-0.912)	0.827 (0.782-0.864)	0.823 (0.783-0.860)	0.777 (0.721-0.824)	0.905 (0.866-0.934)	0.131 (0.110-0.153)
**RF**	**0.913 (0.893-0.937)**	**0.842 (0.810-0.871)**	**0.910 (0.866-0.941)**	**0.796 (0.748-0.836)**	**0.825 (0.788-0.858)**	**0.755 (0.700-0.802)**	**0.928 (0.891-0.953)**	**0.111 (0.095-0.128)**
GB	0.905 (0.888-0.930)	0.833 (0.800-0.862)	0.933 (0.892-0.959)	0.765 (0.716-0.808)	0.821 (0.784-0.854)	0.732 (0.678-0.781)	0.943 (0.908-0.965)	0.146 (0.121-0.173)
XGBoost	0.906 (0.884-0.931)	0.839 (0.806-0.867)	0.937 (0.897-0.962)	0.771 (0.722-0.813)	0.826 (0.790-0.859)	0.739 (0.684-0.786)	0.947 (0.913-0.968)	0.131 (0.109-0.155)
SVM	0.905 (0.880-0.931)	0.841 (0.808-0.869)	0.915 (0.871-0.945)	0.789 (0.742-0.830)	0.824 (0.788-0.858)	0.750 (0.695-0.798)	0.931 (0.894-0.955)	0.119 (0.102-0.137)
MLP	0.891 (0.864-0.912)	0.817 (0.782-0.847)	0.951 (0.914-0.972)	0.724 (0.673-0.770)	0.809 (0.771-0.843)	0.704 (0.650-0.753)	0.955 (0.921-0.975)	0.156 (0.141-0.171)

Abbreviations: LR, Logistic regression; DT, Decision Tree; RF, Random Forest; GB, Gradient Boosting; XGBoost, eXtreme Gradient Boosting; SVM, Support Vector Machine; MLP, Multi-Layer Perceptron; PPV, positive predictive value; NPV, negative predictive value; CI, confidence interval.

Table S5. Performance of the reduced Random Forest model based on routinely available CBC parameters in the validation cohort.

Model	AUC	Accuracy	Sensitivity	Specificity	F1 score	PPV	NPV
Reduced RF	0.905 (95% CI: 0.884-0.929)	0.839 (95% CI: 0.806-0.867)	0.951 (95% CI: 0.914-0.972)	0.762 (95% CI: 0.712-0.805)	0.828 (95% CI: 0.793-0.861)	0.734 (95% CI: 0.680-0.781)	0.957 (95% CI: 0.925-0.976)

Abbreviations: RF, Random Forest; AUC, area under the receiver operating characteristic curve; PPV, positive predictive value; NPV, negative predictive value; CI, confidence interval.

Table S6. Performance of machine-learning models for distinguishing thalassemia from non-thalassemia anemia after exclusion of normal controls.

Model	Accuracy (95% CI)	Sensitivity (95% CI)	Specificity (95% CI)	F1 score (95% CI)	PPV (95% CI)	NPV (95% CI)
LR	0.781 (0.739-0.817)	0.937 (0.897-0.962)	0.607 (0.538-0.672)	0.818 (0.780-0.853)	0.726 (0.671-0.774)	0.897 (0.835-0.938)
DT	0.759 (0.717-0.798)	0.933 (0.892-0.959)	0.567 (0.498-0.634)	0.803 (0.763-0.839)	0.705 (0.651-0.754)	0.884 (0.817-0.928)
**RF**	0.816 (0.776-0.850)	0.897 (0.850-0.930)	0.726 (0.661-0.783)	0.837 (0.799-0.870)	0.784 (0.730-0.830)	0.864 (0.804-0.908)
GB	0.807 (0.766-0.841)	0.910 (0.866-0.941)	0.692 (0.625-0.751)	0.832 (0.795-0.865)	0.766 (0.711-0.813)	0.874 (0.814-0.917)
XGBoost	0.785 (0.744-0.822)	0.946 (0.908-0.969)	0.607 (0.538-0.672)	0.823 (0.786-0.856)	0.728 (0.674-0.776)	0.910 (0.850-0.948)
SVM	0.797 (0.756-0.833)	0.897 (0.850-0.930)	0.687 (0.619-0.747)	0.823 (0.784-0.858)	0.760 (0.705-0.808)	0.857 (0.795-0.903)
MLP	0.759 (0.717-0.798)	0.951 (0.914-0.972)	0.547 (0.478-0.615)	0.806 (0.769-0.841)	0.700 (0.646-0.749)	0.909 (0.845-0.948)

Abbreviations: LR, Logistic regression; DT, Decision Tree; RF, Random Forest; GB, Gradient Boosting; XGBoost, eXtreme Gradient Boosting; SVM, Support Vector Machine; MLP, Multi-Layer Perceptron; PPV, positive predictive value; NPV, negative predictive value; CI, confidence interval.

Table S7. Performance of the RF model and conventional screening indices at a matched sensitivity level of approximately 97.3%.

Screening method	Specificity	PPV	NPV

RF	0.731	0.714	0.975
MCV/MCH rule	0.647	0.656	0.972
Mentzer index	0.368	0.515	0.952
RDWI	0.285	0.484	0.939
England and Fraser index	0.285	0.484	0.939
Sirachainan index	0.288	0.485	0.939

Abbreviations: RF, Random Forest; PPV, positive predictive value; NPV, negative predictive value; RDWI, red cell distribution width index.
